# Opportunities and limitations of genomics for diagnosing bedaquiline-resistant tuberculosis: a systematic review and individual isolate meta-analysis

**DOI:** 10.1016/S2666-5247(23)00317-8

**Published:** 2024-01-09

**Authors:** Camus Nimmo, Neda Bionghi, Matthew J Cummings, Rubeshan Perumal, Madeleine Hopson, Shamim Al Jubaer, Kogieleum Naidoo, Allison Wolf, Barun Mathema, Michelle H Larsen, Max O’Donnell

**Affiliations:** Francis Crick Institute, London, UK; Department of Medicine, Columbia University Irving Medical Center and New York-Presbyterian Hospital, New York, NY, USA; Division of Pulmonary, Allergy, and Critical Care Medicine, Department of Medicine, Columbia University Irving Medical Center, New York, NY, USA; CAPRISA-MRC HIV-TB Pathogenesis and Treatment Research Unit, Durban, South Africa, Division of Pulmonology and Critical Care, Department of Medicine, Inkosi Albert Luthuli Central Hospital, University of KwaZulu-Natal, Durban, South Africa; Department of Medicine, Columbia University Irving Medical Center and New York-Presbyterian Hospital, New York, NY, USA; Department of Microbiology and Immunology, Albert Einstein College of Medicine, Bronx, NY, USA; CAPRISA-MRC HIV-TB Pathogenesis and Treatment Research Unit, Durban, South Africa; Division of Pulmonary, Allergy, and Critical Care Medicine, Department of Medicine, Columbia University Irving Medical Center, New York, NY, USA; Department of Epidemiology, Mailman School of Public Health, Columbia University Irving Medical Center, New York, NY, USA; Department of Microbiology and Immunology, Albert Einstein College of Medicine, Bronx, NY, USA; Division of Pulmonary, Allergy, and Critical Care Medicine, Department of Medicine, Columbia University Irving Medical Center, New York, NY, USA, CAPRISA-MRC HIV-TB Pathogenesis and Treatment Research Unit, Durban, South Africa, Division of Pulmonology and Critical Care, Department of Medicine, Inkosi Albert Luthuli Central Hospital, University of KwaZulu-Natal, Durban, South Africa

## Abstract

**Background:**

Clinical bedaquiline resistance predominantly involves mutations in *mmpR5* (*Rv0678*). However, *mmpR5* resistance-associated variants (RAVs) have a variable relationship with phenotypic *Mycobacterium tuberculosis* resistance. We did a systematic review to assess the maximal sensitivity of sequencing bedaquiline resistance-associated genes and evaluate the association between RAVs and phenotypic resistance, using traditional and machine-based learning techniques.

**Methods:**

We screened public databases for articles published from database inception until Oct 31, 2022. Eligible studies performed sequencing of at least *mmpR5* and *atpE* on clinically sourced *M tuberculosis* isolates and measured bedaquiline minimum inhibitory concentrations (MICs). A bias risk scoring tool was used to identify bias. Individual genetic mutations and corresponding MICs were aggregated, and odds ratios calculated to determine association of mutations with resistance. Machine-based learning methods were used to define test characteristics of parsimonious sets of diagnostic RAVs, and *mmpR5* mutations were mapped to the protein structure to highlight mechanisms of resistance. This study was registered in the PROSPERO database (CRD42022346547).

**Findings:**

18 eligible studies were identified, comprising 975 *M tuberculosis* isolates containing at least one potential RAV (mutation in *mmpR5*, *atpE*, *atpB*, or *pepQ*), with 201 (20⋅6%) showing phenotypic bedaquiline resistance. 84 (29⋅5%) of 285 resistant isolates had no candidate gene mutation. Sensitivity and positive predictive value of taking an any mutation approach was 69% and 14%, respectively. 13 mutations, all in *mmpR5*, had a significant association with a resistant MIC (adjusted p<0⋅05). Gradient-boosted machine classifier models for predicting intermediate or resistant and resistant phenotypes both had receiver operator characteristic c statistic of 0⋅73 (95% CI 0⋅70–0⋅76). Frameshift mutations clustered in the α1 helix DNA-binding domain, and substitutions in the α2 and α3 helix hinge region and in the α4 helix-binding domain.

**Interpretation:**

Sequencing candidate genes is insufficiently sensitive to diagnose clinical bedaquiline resistance, but where identified, some mutations should be assumed to be associated with resistance. Genomic tools are most likely to be effective in combination with rapid phenotypic diagnostics. This study was limited by selective sampling in contributing studies and only considering single genetic loci as causative of resistance.

**Funding:**

Francis Crick Institute and National Institute of Allergy and Infectious Diseases at the National Institutes of Health.

## Introduction

Bedaquiline is a WHO group A drug that is included in all multidrug-resistant (MDR) tuberculosis treatment and is a core part of new 6-month all-oral regimens for MDR tuberculosis alongside pretomanid and linezolid.^1^ It is also included in new clinical trials for short-course treatment of drug-susceptible tuberculosis (NCT03338621).^2^ Bedaquiline was licensed for clinical use in 2012, with preclinical laboratory studies reporting resistance through mutations in the *atpE* gene encoding a subunit of the target ATP pump.^3^ It was not until 4 years later that the main mechanism of clinical bedaquiline resistance was identified— mutations in the *mmpR5* (*Rv0678*) efflux pump negative repressor, which leads to overexpression of the MmpL5 efflux pump.^4^ Many resistance-associated variants (RAVs) throughout *mmpR5* have now been reported, most of which are individually rare, and have a variable relationship to phenotypic resistance.^5^ In particular, many putative *mmpR5* RAVs are associated with small increases in bedaquiline minimum inhibitory concentration (MIC), which fall near or under the critical concentration for defining resistance.^6^ Understanding bedaquiline resistance is important as it is now reported in about 3% of all MDR-tuberculosis cases globally,^7^ rising to up to 11% in some cohorts,^8^ and over 50% among patients for whom bedaquiline-based regimens have failed.^9^

The absence of a defined genetic hotspot for bedaquiline resistance makes designing a molecular diagnostic that targets a small number of high-confidence mutations in a particular genomic region, such as Xpert MTB/RIF or MTB/XDR assays, challenging. If genotypic–phenotypic correlations can be established, then targeted or whole-genome sequencing approaches could be used to diagnose bedaquiline resistance. The current version of the WHO mutation catalogue contains six mutations associated with resistance, and 13 that have been given an interim association pending further data.^10^ However, the number of *Mycobacterium tuberculosis* isolates eligible for inclusion was limited by the exclusion of heteroresistant mutations at frequency <75% (which are common in *mmpR5*) from the main analysis.

In this study, we collate all genotypic and phenotypic bedaquiline resistance data reported from clinical isolates upto Oct 31, 2022, including the more than 100 bedaquiline-resistant isolates from the CRyPTIC study, with almost twice the number of isolates with *mmpR5* mutations included of the most recent analysis.^5^ We use a more inclusive method than the WHO mutation catalogue, incorporating heteroresistant mutations to evaluate genotype–phenotype correlations for putative bedaquiline-resistance conferring variants and assess how effective sequencing of resistance-associated genes would be for the identification of bedaquiline resistance, in keeping with the current positioning by WHO of targeted next-generation sequencing as aninitial drug-resistance test.^11^ Unlike the WHO catalogue we exclude variants not derived from clinical samples. We evaluate the association of individual mutations with resistance using standard statistical methods, as well as machine-based learning methods, and map specific *mmpR5* mutations to the protein structure to highlight mechanisms of bedaquiline resistance.

## Methods

### Search strategy and study selection

The study protocol for this systematic review and individual isolate meta-analysis was developed using the PRISMA guidelines, and was registered in PROSPERO (CRD42022346547). We searched for primary studies in electronic databases PubMed, Cochrane, and Embase that were published from database inception to Oct 31, 2022. We conducted manual journal and article searches in the *American Journal of Respiratory and Critical Care Medicine* and the *New England Journal of Medicine*. References from studies were also screened for inclusion. Our search strategy included the terms “bedaquiline resistance”, “bedaquiline”, “tuberculosis”, “RAVs”, “drug-resistant tuberculosis”, “Rv0678”, “atpE”, “pepQ”, “mmpL5”, “mmpR”, “Rv2535c”, and “Rv1305”. Concepts were exploded to include all MeSH subheadings and reveal any further articles.

Inclusion required that a study (1) measure the number of clinical *M tuberculosis* isolates or patients with bedaquiline resistance via MICs with a technique for which a WHO-approved critical concentration (CC) exists (ie, MGIT or 7H11 agar), or using broth microdilution as was used for the CRyPTIC study and a good evidence base behind the epidemiological cutoff;^12^ and (2) perform sequencing of at least the *mmpR5* and *atpE* genes. Studies that were not in English (or translated into English), had CCs not consistent with the WHO CCs,^6^ were irrelevant to the study question, used strictly in vitro studies (ie, *M tuberculosis* isolates not obtained from patients), were case reports or case series with less than or equal to three patients, or for which the full text was unavailable, were excluded. PRISMA was used for reporting search results and final studies included in the systematic review. The following definitions were used: MDR tuberculosis was defined as resistance to isoniazid and rifampicin; rifampicin-resistant tuberculosis was defined as resistance to at least rifampicin; and clinical *M tuberculosis* isolates were samples obtained from patients capable of undergoing MIC testing.

### Data extraction and definitions

Two reviewers (NB and MH) independently screened citations using a standardised form with predefined inclusion and exclusion criteria, and extracted data using a standardised data collection form. In cases of disagreement in screening or data abstraction, the disagreementwas referred to a third reviewer (MO) to resolve differences and achieve consensus. Duplicate studies of the same cohort were excluded, and if multiple publications included overlapping cohort data, the most comprehensive study was included. We recorded available information on both isolates and patients. Data included MICs for bedaquiline, numbers of isolates or patients with bedaquiline resistance categorised by pretreatment or during treatment, RAVs identified, and MICs associated with all identified RAVs. Following article retrieval, we applied the US Preventive Services Task Force criteria for evaluation of internal validity of studies to all included studies.^13^ No studies were excluded due to limitations in the validity as assessed by this tool. Publication bias was evaluated using funnel plots and methodological quality was assessed using an established tool.^14^

Candidate genes for bedaquiline resistance were deemed to be *atpE*, *mmpR5* (*Rv0678*), *pepQ*, and *atpB*. Potential RAVs were non-synonymous single-nucleotide variants (SNVs), all indels, and any mutations 100 bp upstream of the start codon, thus probably including the promoter region. All insertions and deletions at a given position were grouped together, as reported in the CRyPTIC study (the largest contributor of isolates). Previous studies have assumed that all frameshift indels will lead to a loss of function.^15^ Variants were included if present at a frequency of 5% or more. Due to the area of technical uncertainty around the CC for bedaquiline, we deemed isolates with MICs greater than the CC to be resistant, those with MICs at the CC to be intermediate, and those with MICs below to be susceptible. CCs were 1 μg/mL in MGIT and 0⋅25 μg/mL on 7H11 agar and broth microdilution plate.^6,12^

### Data analysis

To calculate the sensitivity of genomic methods for resistance, all mutations in resistant (or intermediate or resistant) isolates that had been routinely phenotyped were incorporated (excluding studies where phenotyping was only performed on isolates with RAVs). To calculate the positive predictive value (PPV) of mutations for resistance, all isolates that were routinely sequenced were included (excluding studies where only phenotypically resistant isolates were sequenced). Methods used to determine resistance for each included study are listed in appendix 1 (p 3).

The association of individual variants with resistance, as well as negative predictive value (NPV) and specificity, was calculated using a dataset of all extracted isolates from included studies (ie, all those with resistance or mutations in candidate genes) and the curated reuse CRyPTIC dataset of 12 288 isolates, which is the format the CRyPTIC consortium provides as a curated repository, which subsequent projects are advised to use.^16^ Isolates with more than one mutation across all candidate genes were excluded from the association of individual variants with resistance. We used previously established methods to calculate the odds ratio (OR), likelihood ratio, and p value for the association of putative RAVs with resistance using Stata, version 17.^15^ We corrected SNVs, but not indels, for the false discovery rate using the Benjamini-Hochberg procedure. Indels are annotated as the nucleotide position followed by “_indel”, whereas SNVs are annotated with amino acid substitution. We used a significance level of p<0⋅05.

### Machine learning models to determine a parsimonious set of diagnostic RAVs

To identify a parsimonious set of RAVs that might enhance diagnosis of phenotypic bedaquiline resistance, we performed feature selection using gradient-boosted machine classifier models to identify RAVs most important in classification of phenotypic resistance (*mlr, xgboost* R packages). For each resistance phenotype (resistant and resistant intermediate), classifier models were applied to RAVs that occurred with an absolute frequency of three or more. From each classifier model, we identified the five and ten most important RAVs based on their respective split–gain values. These values reflect improvement in classification accuracy gained when the feature in question (ie, RAV) is added to the model decision tree, with higher values indicating greater importance. Discriminatory performance of parsimonious RAV sets for prediction of each resistance phenotype (dependent variable) was evaluated by generating multi-variable logistic regression models that included the five and ten most important RAVs as independent variables. As several RAVs predicted both resistance phenotypes perfectly (ie, complete separation was present in the dataset), we applied the likelihood penalty developed by Firth to our logistic models using the *logistf* R package.^17^ For each Firth-penalised logistic model, we generated receiver operating characteristic (ROC) curves and computed the area under each curve (AUROC) with associated 95% CIs, the latter of which were derived using 10 000 bootstrapped replicates.

To minimise classifier model overfitting, we split our sample of isolates into random derivation (70%; n=682) and validation (30%; n=293) sets (*caTools* R package). For each resistance phenotype, parsimonious RAV sets (ie, the five and ten most important in each classifier model) were determined exclusively in the derivation set, with discriminatory performance of each set determined in the derivation and validation sets independently. Full details are in appendix 1 (p 2).^17^

### Mapping RAVs onto a three-dimensional MmpR5 protein model

The crystal structure of MmpR5 was reported by Radhakrishnan and colleagues^18^ and deposited as 4NB5. The MmpR5 4NB5 file was rendered in PyMol, version 2.0, and bedaquiline RAVs were depicted as stick structures.

### Role of the funding source

The funders had no role in study design, data collection, data analysis, data interpretation, or writing of the report.

## Results

**Figure 1:**
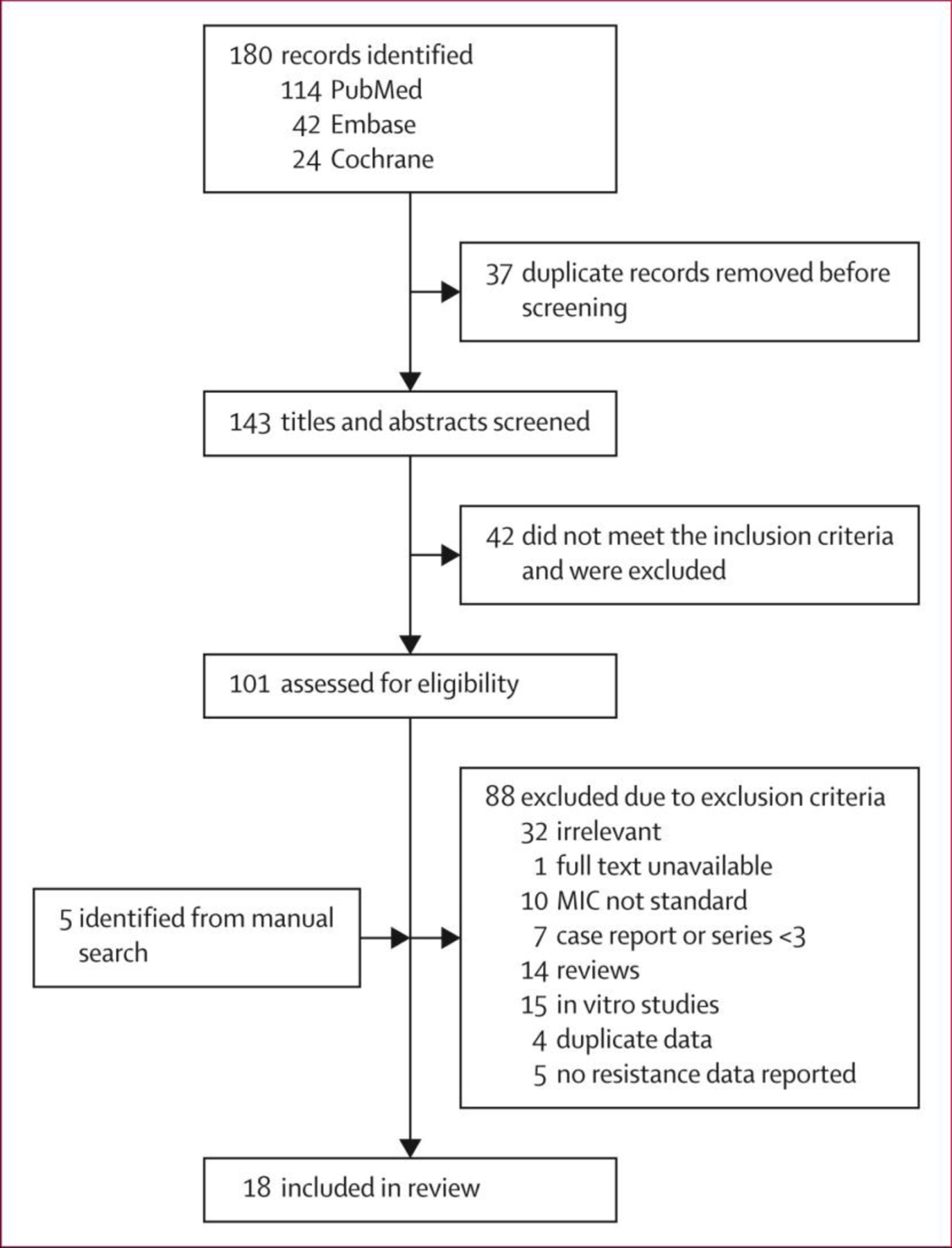
Study profile MIC=minimum inhibitory concentrations.

18 studies met inclusion criteria (figure 1; appendix 1 p 3). 11 studies were assessed as low risk of bias and seven as moderate risk of bias (appendix 1 p 4). These studies yielded 975 independent isolates containing one or more mutation in at least one candidate resistance-associated gene (ie, potential RAV), of which 545 (55⋅9%) had one or more mutation in *mmpR5*. Phenotypic resistance testing showed an intermediate MIC (MIC=CC) for 132 isolates and resistant MIC (MIC>CC) for 201 isolates with candidate gene mutations. An additional 195 intermediate and 84 resistant isolates were identified with no mutations in candidate genes, giving a total of 1254 included isolates of which 274 (21⋅9%) were fully bedaquiline resistant. 943 (75⋅2%) of 1254 isolates were derived from the CRyPTIC study.^16^ All isolates included in the study are reported in appendix 2. Seven isolates had mutations in more than one candidate gene (appendix 1 p 5).

To determine the maximum achievable sensitivity for phenotypic bedaquiline resistance, our first approach was to assume that any mutation (non-synonymous SNV, promoter mutation, or indel) in any candidate gene(*mmpR5*, *atpE*, *atpB*, or *pepQ*), or *mmpR5* alone, might cause resistance. Sensitivity of *mmpR5* mutations alone was close to 50% for intermediate or resistant isolates, with inclusion of *atpE*, *atpB*, and *pepQ* increasing sensitivity by approximately 4% (table 1). Sensitivity for full resistance was higher at over 65% for both methods (*mmpR5* or all candidate genes). PPV was highest when using any *mmpR5* mutation to predict an intermediate or resistant phenotype at 49⋅4%. It was lower for *atpE* (47⋅4%), and less than 10% for *atpB* and *pepQ* genes, which contained many variants not associated with resistance (table 1).

We then evaluated the specificity and NPV of inferring resistance from the methods with sensitivity approaching at least 50%: any mutation in any candidate gene, or from *mmpR5* only (table 1). We used a simulated highly drug-resistant population combining all isolates from included studies and combined this with the entire CRyPTIC reuse database (including an additional 11 135 isolates, which were bedaquiline susceptible and were wild type in candidate genes). The CRyPTIC dataset includes a significant number of drug-resistant isolates (39% rifampicin resistant).^19^ Specificity was high ( 94%), as was NPV (>97%), consistent with the fact that bedaquiline resistance is rare in this dataset (<3% with intermediate or resistant phenotype).

We modelled the performance of these tests at varying prevalence of resistance in the test population (table 2). This shows that if population prevalence of bedaquiline resistance increased, PPV would rise and NPV would fall.

Across all candidate genes there were 62 variants that occurred independently of other candidate gene variants at least three times and were selected for further evaluation (appendix 1 pp 6–9). 24 variants had a significant association with an intermediate or resistant MIC: 23 in *mmpR5* and one in *atpB* (table 3; appendix 1 pp 6–7). Most had a strong association (OR >10), except *mmpR5* 192_indel, which had a significant association but weaker strength (OR 6⋅58). Only one *atpE* variant, Glu61Asp, was recorded independently at least three times. It was associated with one susceptible, one intermediate, and one resistant phenotype, although the association was not statistically significant (appendix 1 pp 6–7). Restricting the analysis to only resistant MICs, 13 variants had a significant association, all in *mmpR5* and all with a strong association except *mmpR5* 192_indel (OR 3⋅61; table 3, appendix 1 pp 8–9).

**Figure 2:**
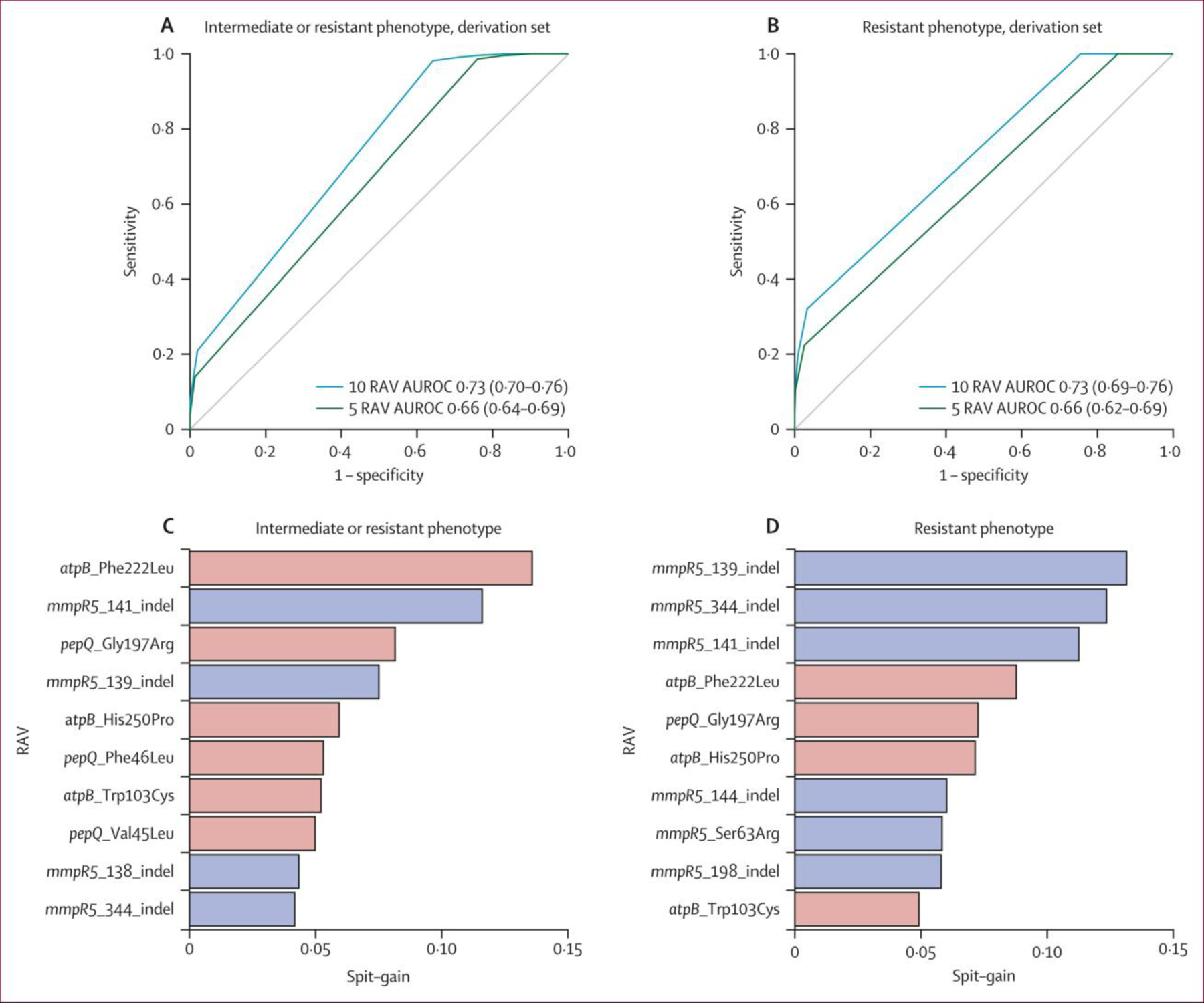
Discriminatory performance of gradient-boosted machine classifier models for RAV-based prediction of resistant and resistant or intermediate phenotypes ROC curves for RAV-based prediction in the derivation cohort for intermediate or resistant phenotype (A) and resistant phenotype (B). AUROC presented with 10 000 bootstrap-derived 95% CIs. The ten most important RAVs shown for intermediate or resistant phenotype (C) and resistant phenotype (D). Orange bars indicate association with susceptibility to bedaquiline and blue bars indicate non-susceptibility to bedaquiline. ROC=receiver operating characteristic. RAV=resistance-associated variant. AUROC=area under the receiver operating characteristic curve.

Across all isolates, 71 RAVs that occurred with a frequency of three or more (including occurrences that were not independent of other candidate gene variants) were included in each gradient-boosted machine classifier model. For the intermediate or resistant phenotype, AUC-ROC in the derivation set was 0⋅73 (95% CI 0⋅70–0⋅76) and 0⋅66 (0⋅64–0⋅69) for the ten and five most important RAVs, respectively (figure 2A). For the resistant phenotype, AUC-ROC in the derivation set was 0⋅73 (0⋅69–0⋅76) and 0⋅66 (0⋅62–0⋅69) for the ten and five most important RAVs, respectively (figure 2B). Ranked importance of RAVs in predicting susceptible versus resistant and resistant or intermediate phenotypes in each classifier model is presented in figure 2C and 2D. For both resistance phenotypes, findings were similar in the validation sets (appendix p 10).

The mutations identified by machine learning to be most important for classification of resistance were indels in *mmpR5*, around the hotspot at nucleotides 138–144 as well as positions 198 and 344. These were also strongly associated with resistance in the individual mutation analysis (table 3). The Ser63Arg substitution was also strongly associated with resistance. The remaining ten most important mutations were predictive of susceptibility rather than intermediate or resistant phenotypes.

**Figure 3:**
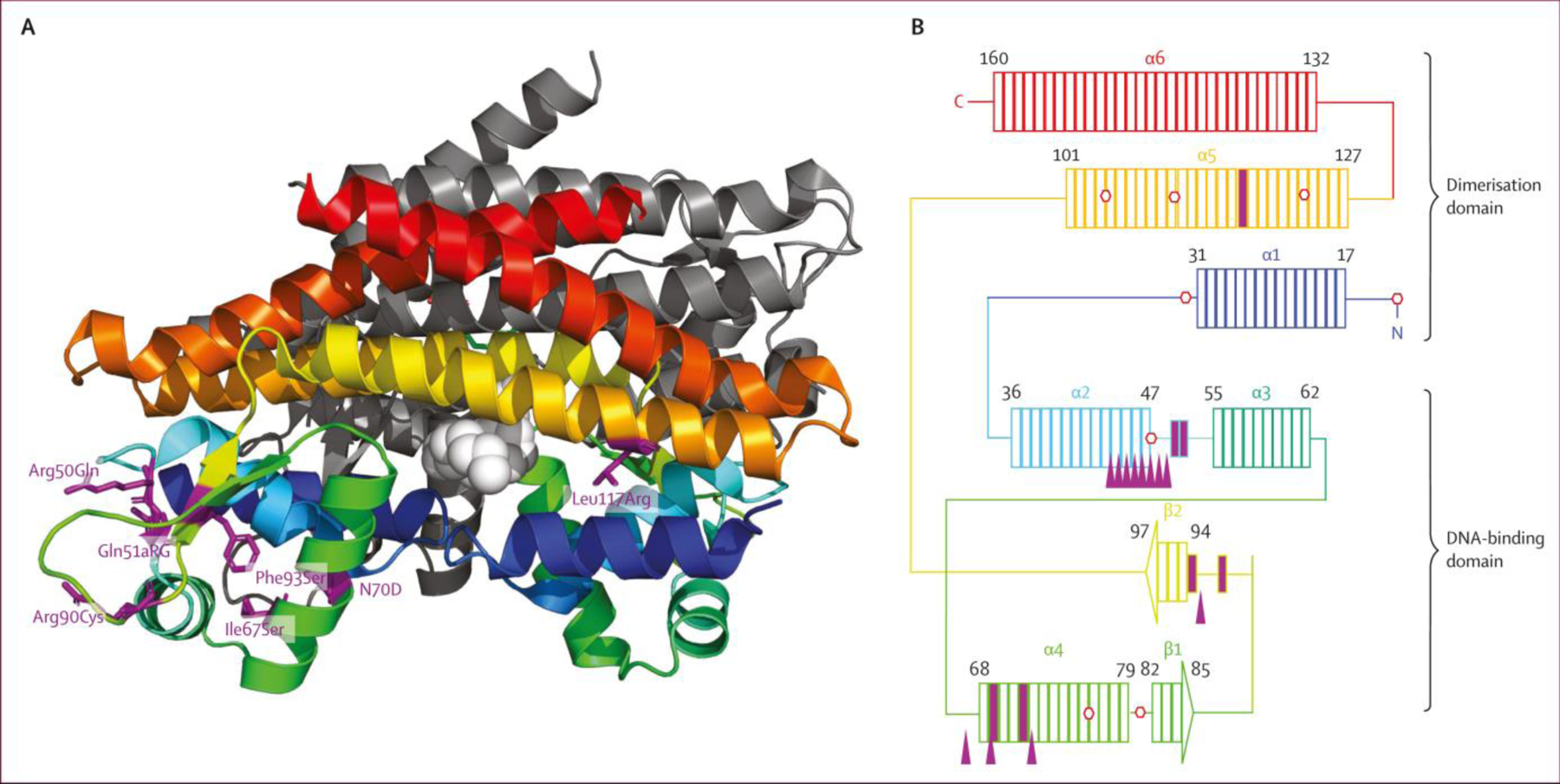
Mapping of bedaquiline resistance-associated variants to the secondary structure of MmpR5 (A) The predicted structure of MmpR5 (PDB 4NB5) is two homodimers. One homodimer is rendered in colour with a rainbow progression (blue, green, yellow, orange, and red) from the N terminal with the remaining homodimer in grey. The fatty acid ligand described in Radhakhrishnan et al^18^ is rendered in light grey spheres. Amino acid residues that are sites of amino acid substitutions (table 3) are purple stick structures. (B) Schematic of an MmpR5 monomer. Rectangles represent amino acids of MmpR5 and colouring corresponds to (A). The DNA-binding domain comprises α helices 2, 3, and 4, as well as β sheets 1 and 2. The dimerisation domain is comprised of α helices 1, 5, and 6. Amino acid substitutions are indicated by solid purple rectangles. Stop codons in reading frames 2 or 3 are depicted as red hexagons. Indels (table 1) are shown as purple filled triangles.

A representation of the MmpR5 protein structure is shown in figure 3. Eight of the 14 indels in *mmpR5* associated with bedaquiline resistance map to a 13-bp stretch (nucleotides 132–144; codons 44–49). These indels are in the coding region for the α2 helix (part of the DNA-binding domain), which correlates with previous reports: frameshift mutations at nucleotides 138–144 and 212–216 were the mutations most frequently associated with bedaquiline resistance in a 2020 review,^20^ and codons 46–49 and codon 67 were found in 87 (43⋅7%) of 199 South Africa patient isolates showing bedaquiline resistance in a later report.^21^ Such homopolymer indels have been previously implicated in drug resistance in the *glpK* gene.^22^ *mmpR5* has three homopolymer regions of 6 bp (nucleotides 139–144), 7 bp (nucleotides 192–198), and 5 bp (nucleotides 212–216).

DNA-binding domain mutations Arg50Gln and Gln51Arg are in the unstructured region between the α2 and α3 helices.^18^ At Arg50Gln or Gln51Arg there are substitutions of glutamine and arginine. Comparatively, glutamine is positively charged or hydrophilic and larger than the polar or uncharged Arg. Codons 50 and 51 are in the seven amino acids between α2 and α3, so substitutions of an amino acid with a different size and charge could alter the hinge between these helices, which are part of the overall DNA-binding domain. The α4 helix is predicted to bind DNA and there are two amino acid substitution mutations Ile67Ser and Asn70Asp associated with.^18^ For Ile67Ser the smaller polar amino acid Ser with a reactive hydroxyl group is substituted for the larger and very hydrophobic amino acid Ile. Also, in the α4 helix, an Asn70Asp mutation substitutes similarly sized amino acids with a negatively charged side chain (Asp) for an amino acid with a polar uncharged side chain (Asn). There are three loss of function indel mutations in codons 64, 67, and 71. In the eight codons between the β1 and β2 sheets, this is one loss of function mutation (274_indel) and two amino acid substitutions (Arg90Cys and Phe93Ser). Arg90 is a highly conserved residue in the MarR family of transcriptional regulators. The Arg90Cys mutation is at a key residue and substitutes the smaller hydrophobic amino acid cysteine for Arg, an amino acid with a positively charged side chain. Aromatic side chains of amino acids can interact with non-protein ligands that contain aromatic groups. These aromatic stacking interactions have been proposed for Tyr145 and Tyr157 of MmpR5. The Phe93Seraminoacid substitution replaces the large and very hydrophobic Phe withanaromatic ring for the much smaller uncharged polar amino acid Ser.

The helices α1, α5, and α6 comprise the dimerisation domain. From table 3, Leu117Arg is the only mutation strongly associated with resistance that is found in the dimerisation region. For Leu117Arg, the smaller and very hydrophobic leucine is replaced by the much larger hydrophilic arginine that has a reactive side chain.

## Discussion

Even using the most sensitive approach possible, where any mutation in any of the genes that have been identified as potentially associated with bedaquiline resistance is assumed to be resistance conferring, only 49–69% of bedaquiline resistance could be identified (compared with 49% quoted by the WHO mutation catalogue using the current mutation list).^10^ However, even in this cohort with a high prevalence of drug resistance (41% of the final cohort were rifampicin resistant), an any mutation candidate gene approach would have a high specificity and NPV (>94%), although this is expected given the low rate of overall bedaquiline resistance in the cohort. The remaining phenotype resistance that is unexplained by currently identified genetic targets might be caused by epigenetic mechanisms. MmpR5 is similar to RND efflux pumps in Gram-negative bacteria, which are known to be regulated by both local and global transcriptional regulators, as well as two-component regulatory systems and post-transcriptional modification.^23^

The sensitivity of genomics to predict bedaquiline resistance is, therefore, less than for the main first-line drugs rifampicin (92%) and isoniazid (90%), and second-line drugs levofloxacin (84%) or moxifloxacin (87%).^24^ It is, however, equivalent to the sensitivity for pyrazinamide (60%),^24^ which has a similar mechanism of resistance with multiple different loss of function mutations. This similarity is important as, to date, rapid molecular diagnostic assays have been successfully designed and implemented for rifampicin, isoniazid, and fluoroquinolone drugs, although not for pyrazinamide. At sensitivities of 40–60%, genomic tests for pyrazinamide and bedaquiline are both substantially below the 80% sensitivity for detecting resistance required by WHO’s proposed target product profiles for drug susceptibility testing at peripheral centres.^25^

Although the current understanding of the genetic basis of bedaquiline resistance does not yet lend itself to a standalone rapid diagnostic, whole-genome sequencing is already used in several countries, suchas the UK and USA, as the primary resistance test, and current work is underway to develop affordable next-generation sequencing that can be used worldwide. Therefore, it is probable that mutations in candidate genes for bedaquiline resistance will be identified, and it is possible that some of these might be strongly associated with resistance. Incorporating heteroresistant mutations, using microtitre plate MICs, and combining other published studies allowed us to identify 21 mutations that had a strong association with intermediate or resistant bedaquiline MICs, and 11 with resistant bedaquiline MICs (similar to the 19 in the current edition of the WHO mutation catalogue, although there are differences due to variations in method).^10^ The majority of these were insertions or deletions (65⋅0% and 61⋅5%, respectively), suggesting that the main mechanism of resistance is loss of gene function. Using machine learning to identify the ten most predictive mutations of phenotype (either susceptible or resistant), several of these mutations (*mmpR5* indels at positions 138–141, 198, and 344) were major contributors. Therefore, where identified by sequencing, these mutations are likely to indicate resistance and phenotypic testing might no longer be required. Meanwhile, most *pepQ* and *atpB* mutations are unlikely to signify resistance.

Further examining the MmpR5 protein, most indel RAVs were located in a homopolymer tract in the α2 helix, which contains the DNA-binding domain. The main substitution RAVs are located in the hinge region between the α2 and α3 helices and the DNA-binding region of the α4 helix.

A limitation to this study was variability in methods used for determining MICs in different settings, although we tried to limit this by including only studies using WHO-approved methods and broth microdilution, which was extensively used by the CRyPTIC consortium. Also, sequencing varied between sequencing of only candidate genes, and whole–genome sequencing. Not all studies sequenced all genes (we required *mmpR5* and *atpE* as a minimum), and their ability to detect low frequency variants differed. Some of the studies included a risk of selection bias as they were based on convenience sampling of stored isolates (often those from patients who did not respond to treatment), rather than systematic population sampling, which might mean that patterns of mutations and resistance are not representative. Although we aimed to make this study as inclusive as possible, it is probable there is a geographical bias in the location of published studies. This bias means that identified mutations and phenotypes might not be representative of the true global patterns, although it is difficult to predict how.

The current critical concentration might be set too high (given the MIC overlap of wild-type and mutated isolates), which means that we might have failed to make statistical associations for clinically important mutations. We did not formally consider the role of heteroresistance, which again might have led to us missing clinically significant mutations that were not identified due to insufficient sequencing depth or were stochastically lost during MIC testing. In our analysis, we only considered the phenotypic implications of individual genomic mutations and, as it is standard with current molecular diagnostics, did not consider potential interaction of genetic background or epigenetic factors. If these factors are important and tools are able to incorporate them in future analyses, then future genomic tests (eg, whole-genome sequencing) might have a better sensitivity than we report.

In conclusion, with our current understanding, it will remain challenging to adequately diagnose bedaquiline resistance using genomics alone. Given the importance of bedaquiline to rifampicin-resistant tuberculosis regimens, establishing adequate access to phenotypic testing must remain a priority. Rapid phenotypic testing methods such as microscopic observation of drug susceptibility assay or reporter phage might have a role to play here. However, some variants are strongly associated with resistance, and we would recommend assuming that such isolates are phenotypically resistant pending confirmatory phenotypic testing. In populations with known low prevalence of phenotypic bedaquiline resistance, the high NPV might permit use of a genomic rule-out test, although as population prevalence rises NPV would fall and PPV would increase to around 87–95% at 25–50% population prevalence, which is not dissimilar to rates currently reported among treatment-experienced patients.

## Supplementary Material

s1

s2

## Figures and Tables

**Table 1:** Sensitivity, positive predictive value, specificity, and negative predictive value of different genomic approaches for determination of bedaquiline intermediate or resistant, or resistant-only isolates

	Intermediate or resistant	Resistant only
**Sensitivity**		
*mmpR5, atpE*, *atpB*, or *pepQ*	310/591 (52⋅5%)	190/276 (68⋅8%)
*mmpR5*	288/591 (48⋅7%)	183/276 (66⋅3%)
**Positive predictive value**		
*mmpR5*, *atpE*, *atpB*, or *pepQ*	250/891 (28⋅1%)	126/891 (14⋅1%)
*mmpR5*	228/462 (49⋅4%)	120/462 (26⋅0%)
*atpB*	13/210 (6⋅2%)	4/210 (1⋅9%)
*atpE*	9/19 (47⋅4%)	7/19 (36⋅8%)
*pepQ*	7/207 (3⋅4%)	1/207 (0⋅5%)
**Specificity**		
*mmpR5*, *atpE*, *atpB*, or *pepQ*	11 135/11 777 (94⋅5%)	11 330/12 104 (93⋅6%)
*mmpR5*	11 542/11 777 (97⋅6%)	11 753/12 104 (97⋅1%)
**Negative predictive value**		
*mmpR5*, *atpE*, *atpB*, or *pepQ*	11 135/11 414 (97⋅6%)	11 330/11 414 (99⋅3%)
*mmpR5*	11 542/11 844 (97⋅5%)	11 753/11 844 (99⋅2%)

Data are n/N (%).

**Table 2:** Modelled PPV or NPV of *mmpR5* or all candidate genes

	*mmpR5*				*mmpR5*, *atpB*, *atpE*, or *pepQ*			
	Intermediate or resistant		Resistant		Intermediate or resistant		Resistant	
	PPV	NPV	PPV	NPV	PPV	NPV	PPV	NPV
0⋅10	68⋅4%	94⋅5%	71⋅8%	96⋅3%	70⋅9%	94⋅9%	54⋅4%	96⋅4%
0⋅25	86⋅7%	85⋅1%	88⋅4%	89⋅6%	87⋅9%	86⋅0%	78⋅2%	90⋅0%
0⋅50	95⋅1%	65⋅5%	95⋅8%	74⋅2%	95⋅6%	67⋅3%	91⋅5%	75⋅0%

All mutations are assumed to confer resistance at a selection of population-resistance prevalences. PPV=positive predictive value. NPV=negative predictive value.

**Table 3:** Candidate gene variants occurring independently in three or more isolates

Gene	Variant	Number with phenotype			Intermediate or resistant phenotype		Resistant phenotype	
		Resistant	Intermediate	Susceptible	p value	Odds ratio (95% CI)	p value	Odds ratio (95% CI)
*mmpR5*	132_indel	0	2	1	0⋅0070*	38⋅5 (3⋅5–426⋅4)	>0⋅9999	0⋅0
*mmpR5*	137_indel	5	0	0	<0⋅0001*	⋅⋅	<0⋅0001*	⋅⋅
*mmpR5*	138_indel	5	7	4	<0⋅0001*	57⋅7 (18⋅9–183⋅1)	<0⋅0001*	19⋅3 (6⋅8–56⋅9)
*mmpR5*	139_indel	7	1	0	<0⋅0001*	⋅⋅	<0⋅0001*	297⋅3 (37⋅4–2485⋅4)
*mmpR5*	140_indel	1	2	1	<0⋅0001*	57⋅7 (6⋅0–558⋅5)	0⋅0890	14⋅2 (1⋅5–137⋅0)
*mmpR5*	141_indel	13	11	11	<0⋅0001*	42⋅0 (21⋅3–89⋅6)	<0⋅0001*	25⋅1 (13⋅1–52⋅7)
*mmpR5*	144_indel	10	1	1	<0⋅0001*	211⋅7 (27⋅8–1672⋅2)	<0⋅0001*	212⋅4 (48⋅0–1009⋅0)
*mmpR5*	192_indel	4	9	38	<0⋅0001*	6⋅6 (3⋅6–12⋅7)	0⋅0290*	3⋅6 (1⋅3–10⋅2)
*mmpR5*	198_indel	6	2	0	<0⋅0001*	⋅⋅	<0⋅0001*	127⋅4 (26⋅1–647⋅6)
*mmpR5*	211_indel	0	2	1	0⋅0070*	38⋅5 (3⋅5–426⋅4)	>0⋅9999	0⋅0
*mmpR5*	274_indel	2	2	1	<0⋅0001*	77⋅0 (8⋅6–694⋅2)	0⋅0050*	28⋅3 (4⋅7–171⋅3)
*mmpR5*	344_indel	6	0	0	<0⋅0001*	⋅⋅	<0⋅0001*	⋅⋅
*mmpR5*	all_del†	1	2	1	<0⋅0001*	57⋅7 (6⋅0–558⋅5)	0⋅0890	14⋅2 (1⋅5–137⋅0)
*mmpR5*	Glu21Asp	2	1	0	<0⋅0001*	⋅⋅	0⋅0020	84⋅9 (7⋅7–946⋅1)
*mmpR5*	Arg50Gln	2	1	0	<0⋅0001*	⋅⋅	0⋅0020	84⋅9 (7⋅7–946⋅1)
*mmpR5*	Gln51Arg	1	2	0	<0⋅0001*	⋅⋅	0⋅0670	21⋅2 (1⋅9–235⋅7)
*mmpR5*	Ser63Arg	5	1	0	<0⋅0001*	⋅⋅	<0⋅0001*	212⋅4 (25⋅2–1856⋅0)
*mmpR5*	Ile67Ser	3	0	0	<0⋅0001*	⋅⋅	<0⋅0001*	⋅⋅
*mmpR5*	Asn70Asp	2	1	0	<0⋅0001*	⋅⋅	0⋅0020*	84⋅9 (7⋅7–946⋅1)
*mmpR5*	Arg90Cys	4	6	4	<0⋅0001*	48⋅1 (15⋅3–156⋅3)	<0⋅0001*	17⋅0 (5⋅4–55⋅2)
*mmpR5*	Leu117Arg	1	3	2	<0⋅0001*	38⋅5 (7⋅1–211⋅9)	0⋅1300	8⋅5 (1–73⋅2)
*mmpR5*	Gly121Arg	3	0	0	<0⋅0001*	⋅⋅	<0⋅0001*	⋅⋅
*mmpR5*	Arg123Lys	1	2	2	0⋅0010*	28⋅9 (4⋅8–173⋅9)	0⋅1100	10⋅6 (1⋅2–95⋅6)
*atpB*	Thr166Met	2	5	11	<0⋅0001*	12⋅3 (4⋅8–32⋅0)	0⋅0630	5⋅3 (1⋅2–23⋅3)

Benjamini-Hochberg adjusted p value for significance was 0⋅0011.

* Significant.

† *mmpR5* whole gene deletion.

## Data Availability

All data used for this Article are publicly available at referenced sources. All individual isolate data included in this Article are included in appendix 2 (tab 1).

## Research in context

### Evidence before this study

*Mycobacterium tuberculosis* genome sequencing is established as the standard technique for identification of resistance to first-line drugs, based on the reliable association of specific genetic mutations with phenotypic resistance. Resistance to bedaquiline— a key drug for treating resistant tuberculosis—is most commonly associated with mutations in the *mmpR5* gene. However, associating individual mutations with resistance is challenging as many *mmpR5* mutations do not lead to resistance. As many mutations occur infrequently, current evidence to make confident associations between specific *mmpR5* mutations and resistance is scarce. Also, it is not known how much bedaquiline resistance is unexplained by mutations in *mmpR5* (or other recognised genes that are mutated less frequently).

### Added value of this study

This study takes advantage of a recent increase in the number of studies reporting bedaquiline resistance alongside *M tuberculosis* whole-genome sequencing data, which we identify through a systematic review. Most significantly, the CRyPTIC consortium has published a global dataset of genome and phenotype data, which includes over 100 bedaquiline-resistant strains. Using these new data, we show the maximum current sensitivity of a bedaquiline resistance test would be 69%, with a positive predictive value of 26%. However, there is now sufficient evidence to make associations between some specific *mmpR5* mutations and resistance.

### Implications of all the available evidence

Genomic methods currently lack the sensitivity to be routinely used as a screening or diagnostic tool for identification of bedaquiline resistance. However, there is now sufficient evidence to infer bedaquiline resistance based on the identification of some specific *mmpR5* mutations—eg, if they are identified through routine whole-genome sequencing.
