# Supplementary material for: Opportunities and limitations of genomics for diagnosing bedaquiline-resistant tuberculosis: a systematic review and individual isolate meta-analysis
Source: Lancet Microbe. Author manuscript; Available in PMC 2024 May 6. (PMC11072239; doi:10.1016/S2666-5247(23)00317-8)
Supplement: s1 [file NIHMS1984846-supplement-s1.pdf]

THE LANCET
Microbe

Supplementary appendix

This appendix formed part of the original submission and has been peer reviewed.
We post it as supplied by the authors.

Supplement to: Nimmo C, Bionghi N, Cummings MJ, et al. Opportunities and limitations of genomics for diagnosing bedaquiline-resistant tuberculosis: a systematic review and individual isolate meta-analysis. *Lancet Microbe* 2024. https://doi.org/10.1016/S2666-5247(23)00317-8

Supplementary Material

Opportunities and limitations of genomics for diagnosing bedaquiline-resistant tuberculosis: a systematic review and individual isolate meta-analysis

Camus Nimmo PhD, Neda Bionghi MD, Matthew J. Cummings MD, Rubeshan Perumal PhD, Madeleine Hopson MD, Shamim Al Jubaer, Kogieleum Naidoo PhD, Allison Wolf, Barun Mathema PhD, Michelle H. Larsen PhD, Max O'Donnell MD

Supplementary Methods 2
    Machine learning models to determine a parsimonious set of diagnostic RAVs 2
Supplementary Tables 3
Supplementary Figures 10
Supplementary Material References 11

Supplementary Methods

Search Terms

Search terms used were: “bedaquiline resistance”, “bedaquiline,” “tuberculosis,” “RAVs,” “drug-resistant tuberculosis,” “Rv0678,” “atpE,” “pepQ,” “mmpL5,” “mmpR,” “Rv2535c,” and “Rv1305”.

Machine learning models to determine a parsimonious set of diagnostic RAVs

To identify a parsimonious set of RAVs that may enhance diagnosis of phenotypic bedaquiline resistance, we performed feature selection using gradient-boosted machine classifier models to identify RAVs most important in classification of phenotypic resistance (*mlr, xgboost* R packages). For each resistance phenotype (resistant and resistant-intermediate), classifier models were applied to RAVs that occurred with an absolute frequency ≥3. Select model hyperparameters (learning rate [eta], tree depth [max_depth], and number of trees [nround]) were tuned using 10-fold cross validation, with remaining hyperparameters left at default settings. From each classifier model, we identified the 5 and 10 most important RAVs based on their respective split-gain values. These values reflect improvement in classification accuracy gained when the feature in question (i.e. RAV) is added to the model decision tree, with higher values indicating greater importance. Discriminatory performance of parsimonious RAV sets for prediction of each resistance phenotype (dependent variable) was evaluated by generating multivariable logistic regression models that included the 5 and 10 most important RAVs as independent variables. As several RAVs predicted both resistance phenotypes perfectly (i.e. complete separation was present in the dataset), we applied the likelihood penalty developed by Firth to our logistic models using the *logistf* R package.[1] For each Firth-penalised logistic model, we generated receiver-operating-characteristic (ROC) curves and computed the area under each curve (AUC-ROC) with associated 95% confidence intervals (CI), the latter of which were derived using 10,000 bootstrapped replicates.

To minimise classifier model overfitting, we split our sample of isolates into random derivation (70%; N=682) and validation (30%; N=293) sets (*caTools* R package). For each resistance phenotype, predictive RAV sets (i.e. 5 and 10 most important) were determined exclusively in the derivation set, with discriminatory performance of each set determined in the derivation and validation sets independently.

Supplementary Tables

Study and Primary Location	Primary method(s) of resistance determination	Included for		Number of intermediate/ resistant isolates			Genes sequenced	MIC method
		sensitivity analysis	PPV analysis	Total	Geno	Pheno		
**Andres 2019[2] (Germany)**	Phenotype	x		10	10	10	*atpE, mmpR5, mmpS5-L5, pepQ*	MGIT
**Chesov 2022[3] (Moldova)**	Phenotype and genotype	x	x	9	9	9	WGS	MGIT
**Conradie 2020[4] (South Africa)**	Phenotype			1	1	1	WGS	MGIT
**Cryptic Consortium 2022[5] (Global)**	Phenotype and genotype	x	x	943	716	353	WGS	Microtitre
**Ghodousi 2019[6] (Pakistan)**	Phenotype and genotype	x	x	8	8	8	WGS	MGIT
**Ismail 2018[7] (South Africa)**	Phenotype and genotype	x	x	8	6	8	WGS	Microtitre
**Ismail 2022[8] (South Africa)**	Phenotype	x		70	70	69	WGS	Microtitre
**Klopper 2020[9] (South Africa)**	Genotype		x	1	1	1	WGS	MGIT
**Liu 2021[10] (China)**	Phenotype and genotype	x	x	11	8	11	*atpE, mmpR5, pepQ*	Microtitre
**Martinez 2018[11] (Australia)**	Phenotype and genotype	x	x	14	14	3	WGS	Microtitre
**Nimmo 2020[12] (South Africa)**	Genotype		x	21	21	17	WGS	7H11
**Peretokina 2020[13] (Russia)**	Phenotype and genotype	x	x	23	22	8	*mmpR5, atpE, atpB, pepQ*	7H11
**Saeed 2022[14] (Pakistan)**	Phenotype and genotype	x	x	20	9	20	WGS	Microtitre
**Torrea 2015[15] (Global)**	Phenotype and genotype	x	x	20	19	19	*mmpR5*	MGIT
**Veziris 2017[16] (France)**	Phenotype	x		4	3	4	*mmpR5, atpE*	7H11
**Villellas 2017[17] (Global)**	Phenotype and genotype	x	x	22	20	11	*mmpR5, atpE, pepQ*	7H11
**Wu 2021[18] (China)**	Phenotype and genotype	x	x	33	21	27	*mmpR5, atpE, pepQ*	Microtitre
**Yang 2018[19] (South Korea)**	Phenotype and genotype	x	x	32	17	28	*mmpR5, atpE*	Microtitre

Supplementary Table 1. List of studies included and primary method used to determine resistance. Studies that were included in the calculation of sensitivity and positive predictive value (PPV) are indicated. The total number of isolates with resistance, and those with a mutation in a candidate gene (geno) or intermediate/resistant MIC (pheno) are broken down by study.

Study	Assessment Criteria									
	1	2	3	4	5	6	7	8	9	10 Summary
Andres 2019[2]	0	0	0	1	0	0	0	0	1	2 (Low risk)
Chesov 2022[3]	1	1	1	1	1	0	0	0	0	5 (Moderate risk)
Conradie 2020[4]	0	0	0	1	0	0	0	0	0	1 (Low risk)
Cryptic Consortium 2022[5]	1	1	0	1	0	0	0	0	0	3 (Low risk)
Ghodousi 2019[6]	1	1	1	1	0	0	0	0	1	5 (Moderate risk)
Ismail 2018[7]	0	0	1	1	0	0	0	0	1	3 (Low risk)
Ismail 2022[8]	0	0	0	1	0	0	0	0	0	1 (Low risk)
Klopper 2020[9]	1	1	1	0	0	0	0	0	1	4 (Moderate risk)
Liu 2021[10]	1	0	0	0	0	0	0	0	0	1 (Low risk)
Martinez 2018[11]	0	0	1	0	0	0	0	0	0	1 (Low risk)
Nimmo 2020[12]	1	1	1	1	0	0	0	0	1	5 (Moderate risk)
Peretokina 2020[13]	1	1	1	0	0	0	0	0	1	4 (Moderate risk)
Saeed 2022[14]	1	1	1	0	0	0	0	0	1	4 (Moderate risk)
Torrea 2015[15]	1	1	1	0	0	0	0	0	1	4 (Moderate risk)
Veziris 2017[16]	0	0	0	1	0	0	0	0	1	2 (Low risk)
Villellas 2017[17]	1	0	0	1	0	0	0	0	0	2 (Low risk)
Wu 2021[18]	0	0	0	1	0	0	0	0	0	1 (Low risk)
Yang 2018[19]	0	0	1	0	0	0	0	0	0	1 (Low risk)

1. Was the study's target population a close representation of the national DR-TB population? (Yes=0, No=1)
2. Was the sampling frame a true or close representation of the target population? (Yes=0, No=1)
3. Was some form of random selection used to select the sample or was a census undertaken? (Yes=0, No=1)
4. Was the likelihood of non-response bias minimal? (Yes=0, No=1)
5. Were data collected directly from the subjects/isolates (as opposed to a medical records/other sources)? (Yes=0, No=1)
6. Were acceptable case definitions used in the study? (Yes=0, No=1)
7. Were reliable and accepted diagnostic methods for diagnosing bedaquiline resistance utilised? (Yes=0, No=1)
8. Was the same mode of data collection used for all subjects? (Yes=0, No=1)
9. Were the numerator(s) and denominator(s) for the calculation of the proportion of bedaquiline resistance appropriate? (Yes=0, No=1)
10. Summary on the overall risk of study bias (Low risk = 0-3, Moderate risk = 4-6, High risk = 7-9)

Supplementary Table 2. Risk of bias for included studies calculated using a standardised tool.[20]

Source	Gene 1 (mutations)	Gene 2 (mutations)	MIC (method)	Resistance classification
**Chesov 2022**	*mmpR5* (192_indel)	*atpE* (A63P)	2 (MGIT)	Resistant
**Chesov 2022**	*mmpR5* (193_indel, S63G)	*atpE* (E61D, I66M)	2 (MGIT)	Resistant
**Cryptic Consortium 2022**	*mmpR5* (141_indel)	*atpB* (T166M)	1 (microtitre)	Resistant
**Cryptic Consortium 2022**	*pepQ* (G112D)	*atpB* (W103C)	0.25 (microtitre)	Intermediate
**Peretokina 2020**	*mmpR5* (141_indel)	*atpE* (A63P)	16 (MGIT)	Resistant
**Peretokina 2020**	*mmpR5* (138_indel, L114P)	*atpE* (A63V)	8 (MGIT)	Resistant
**Peretokina 2020**	*mmpR5* (288_indel)	*atpE* (A63P)	2 (MGIT)	Resistant

Supplementary Table 3. Isolates with variants in >1 candidate gene.

Gene	Variant	Resistant/ Intermediate	Susceptible	p-value	Odds ratio	LR
mmpR5	132_indel	2	1	0.0070	38.49	38.61
mmpR5	137_indel	5	0	<0.0001	-	-
mmpR5	138_indel	12	4	<0.0001	57.73	58.87
mmpR5	139_indel	8	0	<0.0001	-	-
mmpR5	140_indel	3	1	<0.0001	57.73	58.01
mmpR5	141_indel	24	11	<0.0001	41.99	43.66
mmpR5	144_indel	11	1	<0.0001	211.68	215.53
mmpR5	192_indel	13	38	<0.0001	6.58	6.70
mmpR5	198_indel	8	0	<0.0001	-	-
mmpR5	211_indel	2	1	0.0070	38.49	38.61
mmpR5	274_indel	4	1	<0.0001	76.97	77.47
mmpR5	29_indel	1	2	0.1410	9.62	9.64
mmpR5	344_indel	6	0	<0.0001	-	-
mmpR5	418_indel	1	5	0.2620	3.85	3.85
mmpR5	all_del*	3	1	<0.0001	57.73	58.01
mmpR5	S2R	0	3	>0.9999	0.00	0.00
mmpR5	N4T	0	6	>0.9999	0.00	0.00
mmpR5	M17V	0	3	>0.9999	0.00	0.00
mmpR5	E21D	3	0	<0.0001	-	-
mmpR5	M23V	0	3	>0.9999	0.00	0.00
mmpR5	L40V	0	13	>0.9999	0.00	0.00
mmpR5	R50Q	3	0	<0.0001	-	-
mmpR5	Q51R	3	0	<0.0001	-	-
mmpR5	E55D	0	13	>0.9999	0.00	0.00
mmpR5	A59V	1	2	0.1410	9.62	9.64
mmpR5	S63R	6	0	<0.0001	-	-
mmpR5	I67S	3	0	<0.0001	-	-
mmpR5	N70D	3	0	<0.0001	-	-
mmpR5	M73I	1	2	0.1410	9.62	9.64
mmpR5	I80S	1	3	0.1830	6.41	6.42
mmpR5	L83P	2	1	0.0070	38.49	38.61
mmpR5	R90C	10	4	<0.0001	48.11	48.89
mmpR5	F93L	2	1	0.0070	38.49	38.61
mmpR5	N98D	1	7	0.3330	2.75	2.75
mmpR5	G103S	1	2	0.1410	9.62	9.64
mmpR5	L117R	4	2	<0.0001	38.49	38.73
mmpR5	G121R	3	0	<0.0001	-	-
mmpR5	R123K	3	2	0.0010	28.87	29.00
mmpR5	R134G	1	4	0.2240	4.81	4.82
mmpR5	M139I	2	1	0.0070	38.49	38.61

mmpR5	L142R	2	1	0.0070	38.49	38.61
mmpR5	M146T	2	14	0.1860	2.75	2.75
atpB	G58C	0	5	>0.9999	0.00	0.00
atpB	V87M	0	10	>0.9999	0.00	0.00
atpB	W103C	0	32	0.4060	0.00	0.00
atpB	T166M	7	11	<0.0001	12.25	12.38
atpB	W216L	0	4	>0.9999	0.00	0.00
atpB	F222L	0	56	0.1150	0.00	0.00
atpB	H250P	2	46	>0.9999	0.84	0.84
atpE	E61D	2	1	0.0070	38.49	38.61
pepQ	818_indel	2	6	0.0560	6.41	6.43
pepQ	R7Q	0	9	>0.9999	0.00	0.00
pepQ	V45L	0	22	0.6250	0.00	0.00
pepQ	F46L	0	21	0.6230	0.00	0.00
pepQ	P69L	0	19	>0.9999	0.00	0.00
pepQ	V104L	0	3	>0.9999	0.00	0.00
pepQ	A124V	0	4	>0.9999	0.00	0.00
pepQ	A187E	0	3	>0.9999	0.00	0.00
pepQ	I193T	0	4	>0.9999	0.00	0.00
pepQ	G197R	1	45	0.7280	0.43	0.43
pepQ	A242T	0	6	>0.9999	0.00	0.00
pepQ	V328F	0	4	>0.9999	0.00	0.00

Supplementary Table 4. Associations of variants present ≥3 times in candidate genes with intermediate/resistant phenotype. Significant associations are highlighted. Benjamini-Hochberg adjusted p-values for significance was 0.0011. *deletion of the entire *mmpR5* gene.

Gene	Variant	Resistant	Susceptible/ Intermediate	p-value	Odds ratio	LR
mmpR5	132_indel	0	3	>0.9999	0.00	0.00
mmpR5	137_indel	5	0	<0.0001	-	-
mmpR5	138_indel	5	11	<0.0001	19.30	19.63
mmpR5	139_indel	7	1	<0.0001	297.29	304.75
mmpR5	140_indel	1	3	0.0890	14.16	14.20
mmpR5	141_indel	13	22	<0.0001	25.10	26.25
mmpR5	144_indel	10	2	<0.0001	212.35	220.04
mmpR5	192_indel	4	47	0.0290	3.61	3.65
mmpR5	198_indel	6	2	<0.0001	127.41	130.13
mmpR5	211_indel	0	3	>0.9999	0.00	0.00
mmpR5	274_indel	2	3	0.0050	28.31	28.51
mmpR5	29_indel	0	3	>0.9999	0.00	0.00
mmpR5	344_indel	6	0	<0.0001	-	-
mmpR5	418_indel	0	6	>0.9999	0.00	0.00
mmpR5	all_del*	1	3	0.0890	14.16	14.20
mmpR5	S2R	0	3	>0.9999	0.00	0.00
mmpR5	N4T	0	6	>0.9999	0.00	0.00
mmpR5	M17V	0	3	>0.9999	0.00	0.00
mmpR5	E21D	2	1	0.0020	84.94	85.53
mmpR5	M23V	0	3	>0.9999	0.00	0.00
mmpR5	L40V	0	13	>0.9999	0.00	0.00
mmpR5	R50Q	2	1	0.0020	84.94	85.53
mmpR5	Q51R	1	2	0.0670	21.24	21.31
mmpR5	E55D	0	13	>0.9999	0.00	0.00
mmpR5	A59V	1	2	0.0670	21.24	21.31
mmpR5	S63R	5	1	<0.0001	212.35	216.13
mmpR5	I67S	3	0	<0.0001	-	-
mmpR5	N70D	2	1	0.0020	84.94	85.53
mmpR5	M73I	0	3	>0.9999	0.00	0.00
mmpR5	I80S	0	4	>0.9999	0.00	0.00
mmpR5	L83P	0	3	>0.9999	0.00	0.00
mmpR5	R90C	4	10	<0.0001	16.99	17.22
mmpR5	F93L	0	3	>0.9999	0.00	0.00
mmpR5	N98D	1	7	0.1700	6.07	6.09
mmpR5	G103S	0	3	>0.9999	0.00	0.00
mmpR5	L117R	1	5	0.1300	8.49	8.52
mmpR5	G121R	3	0	<0.0001	-	-
mmpR5	R123K	1	4	0.1100	10.62	10.65
mmpR5	R134G	0	5	>0.9999	0.00	0.00
mmpR5	M139I	1	2	0.0670	21.24	21.31

mmpR5	L142R	1	2	0.0670	21.24	21.31
mmpR5	M146T	0	16	>0.9999	0.00	0.00
atpB	G58C	0	5	0.6290	0.00	0.00
atpB	V87M	0	10	>0.9999	0.00	0.00
atpB	W103C	0	32	>0.9999	0.00	0.00
atpB	T166M	2	16	0.0630	5.31	5.34
atpB	W216L	0	4	>0.9999	0.00	0.00
atpB	F222L	0	56	0.6410	0.00	0.00
atpB	H250P	0	48	0.6300	0.00	0.00
atpE	E61D	1	2	0.0670	21.24	21.31
pepQ	818_indel	1	7	0.1700	6.07	6.09
pepQ	R7Q	0	9	>0.9999	0.00	0.00
pepQ	V45L	0	22	>0.9999	0.00	0.00
pepQ	F46L	0	21	>0.9999	0.00	0.00
pepQ	P69L	0	19	>0.9999	0.00	0.00
pepQ	V104L	0	3	>0.9999	0.00	0.00
pepQ	A124V	0	4	>0.9999	0.00	0.00
pepQ	A187E	0	3	>0.9999	0.00	0.00
pepQ	I193T	0	4	>0.9999	0.00	0.00
pepQ	G197R	0	46	0.6280	0.00	0.00
pepQ	A242T	0	6	>0.9999	0.00	0.00
pepQ	V328F	0	4	>0.9999	0.00	0.00

Supplementary Table 5. Associations of variants present ≥3 times in candidate genes with resistant phenotype. Significant associations are highlighted. Benjamini-Hochberg adjusted p-values for significance was 0.0011. LR = likelihood ratio. *deletion of the entire *mmpR5* gene.

Supplementary Figures

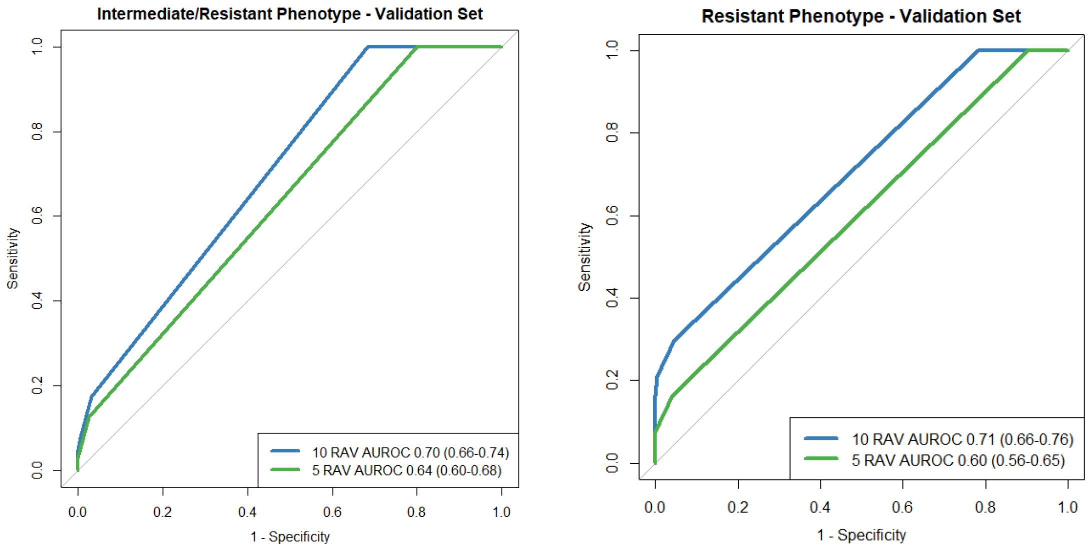

Supplementary Figure 1. Gradient boosted machine classifier model demonstrating ranked importance of RAVs in predicting resistant and resistant/intermediate phenotypes in each classifier model demonstrating AUC-ROC in the validation cohort for (a) intermediate/resistant phenotype and (b) resistant phenotype.

Supplementary Material References

1 Mansournia MA, Geroldinger A, Greenland S, Heinze G. Separation in Logistic Regression: Causes, Consequences, and Control. *Am J Epidemiol* 2017; **187**: 864–70.

2 Andres S, Merker M, Heyckendorf J, *et al.* Bedaquiline-resistant Tuberculosis: Dark Clouds on the Horizon. *Am J Resp Crit Care* 2019; **0**: 1564–8.

3 Chesov E, Chesov D, Maurer FP, *et al.* Emergence of bedaquiline resistance in a high tuberculosis burden country. *European Respir J* 2022; **59**: 2100621.

4 Conradie F, Diacon AH, Ngubane N, *et al.* Bedaquiline, pretomanid and linezolid for treatment of extensively drug resistant, intolerant or non-responsive multidrug resistant pulmonary tuberculosis. *New Engl J Medicine* 2020; **382**: 893–902.

5 The CRyPTIC Consortium. A data compendium associating the genomes of 12,289 Mycobacterium tuberculosis isolates with quantitative resistance phenotypes to 13 antibiotics. *Plos Biol* 2022; **20**: e3001721.

6 Ghodousi A, Rizvi AH, Baloch AQ, *et al.* Acquisition of Cross-Resistance to Bedaquiline and Clofazimine following Treatment for Tuberculosis in Pakistan. *Antimicrob Agents Ch* 2019; **63**: e00915-19.

7 Ismail NA, Omar SV, Joseph L, *et al.* Defining Bedaquiline Susceptibility, Resistance, Cross-Resistance and Associated Genetic Determinants: A Retrospective Cohort Study. *Ebiomedicine* 2018; **28**: 136–42.

8 Ismail NA, Omar SV, Moultrie H, *et al.* Assessment of epidemiological and genetic characteristics and clinical outcomes of resistance to bedaquiline in patients treated for rifampicin-resistant tuberculosis: a cross-sectional and longitudinal study. *Lancet Infect Dis* 2022; **22**: 496–506.

9 Klopper M, Heupink TH, Hill-Cawthorne G, *et al.* A landscape of genomic alterations at the root of a near-untreatable tuberculosis epidemic. *Bmc Med* 2020; **18**: 24.

10 Liu Y, Gao M, Du J, *et al.* Reduced Susceptibility of Mycobacterium tuberculosis to Bedaquiline During Antituberculosis Treatment and Its Correlation With Clinical Outcomes in China. *Clin Infect Dis* 2020; **73**: e3391–7.

11 Martinez E, Hennessy D, Jelfs P, Crighton T, Chen SC-A, Sintchenko V. Mutations associated with in vitro resistance to bedaquiline in Mycobacterium tuberculosis isolates in Australia. *Tuberculosis* 2018; **111**: 31–4.

12 Nimmo C, Millard J, Brien K, *et al.* Bedaquiline resistance in drug-resistant tuberculosis HIV co-infected patients. *Eur Respir J* 2020; **55**: 1902383.

13 Peretokina IV, Krylova LYu, Antonova OV, *et al.* Reduced susceptibility and resistance to bedaquiline in clinical M. tuberculosis isolates. *J Infection* 2020; **80**: 527–35.

14 Saeed DK, Shakoor S, Razzak SA, *et al.* Variants associated with Bedaquiline (BDQ) resistance identified in Rv0678 and efflux pump genes in Mycobacterium tuberculosis isolates from BDQ naïve TB patients in Pakistan. *Bmc Microbiol* 2022; **22**: 62.

15 Torrea G, Coeck N, Desmaretz C, *et al.* Bedaquiline susceptibility testing of Mycobacterium tuberculosis in an automated liquid culture system. *J Antimicrob Chemoth* 2015; **70**: 2300–5.

16 Veziris N, Bernard C, Guglielmetti L, *et al.* Rapid emergence of Mycobacterium tuberculosis bedaquiline resistance: lessons to avoid repeating past errors. *Eur Respir J* 2017; **49**: 1601719.

17 Villellas C, Coeck N, Meehan CJ, *et al.* Unexpected high prevalence of resistance-associated Rv0678 variants in MDR-TB patients without documented prior use of clofazimine or bedaquiline. *J Antimicrob Chemoth* 2017; **72**: 684–90.

18 Wu S-H, Chan H-H, Hsiao H-C, Jou R. Primary Bedaquiline Resistance Among Cases of Drug-Resistant Tuberculosis in Taiwan. *Front Microbiol* 2021; **12**: 754249.

19 Yang JS, Kim KJ, Choi H, Lee SH. Delamanid, Bedaquiline, and Linezolid Minimum Inhibitory Concentration Distributions and Resistance-related Gene Mutations in Multidrug-resistant and Extensively Drug-resistant Tuberculosis in Korea. *Ann Lab Med* 2018; **38**: 563–8.

20 Hoy D, Brooks P, Woolf A, *et al.* Assessing risk of bias in prevalence studies: modification of an existing tool and evidence of interrater agreement. *J Clin Epidemiology* 2012; **65**: 934–9.
